# A simple rat model of mild traumatic brain injury: a device to reproduce anatomical and neurological changes of mild traumatic brain injury

**DOI:** 10.7717/peerj.2818

**Published:** 2017-01-03

**Authors:** Ho Jeong Kim, Soo Jeong Han

**Affiliations:** 1 Department of Rehabilitation Medicine, Seonam Hospital, Ewha Womans University Medical Center, Seoul, Republic of Korea; 2 Department of Rehabilitation Medicine, School of Medicine, Ewha Womans University, Seoul, Republic of Korea

**Keywords:** Concussion, Rat, Device, Methodology

## Abstract

Mild traumatic brain injury typically involves temporary impairment of neurological function. Previous studies used water pressure or rotational injury for designing the device to make a rat a mild traumatic brain injury model. The objective of this study was to make a simple model of causing mild traumatic brain injury in rats. The device consisted of a free-fall impactor that was targeted onto the rat skull. The weight (175 g) was freely dropped 30 cm to rat’s skull bregma. We installed a safety device made of acrylic panel. To confirm a mild traumatic brain injury in 36 Sprague-Dawley rats, we performed magnetic resonance imaging (MRI) of the brain within 24 h after injury. We evaluated behavior and chemical changes in rats before and after mild traumatic brain injury. The brain MRI did not show high or low signal intensity in 34 rats. The mobility on grid floor was decreased after mild traumatic brain injury. The absolute number of foot-fault and foot-fault ratio were decreased after mild traumatic brain injury. However, the difference of the ratio was a less than absolute number of foot-fault. These results show that the device is capable of reproducing mild traumatic brain injury in rats. Our device can reduce the potential to cause brain hemorrhage and reflect the mechanism of real mild traumatic brain injury compared with existing methods and behaviors. This model can be useful in exploring physiology and management of mild traumatic brain injury.

## Introduction

A mild traumatic brain injury (MTBI) or concussion is referred to as a closed head injury, which may be defined as a temporary disturbance in brain function that occurs in a complicated pathophysiological process. In the United States, estimates of up to 3.8 million MTBIs occur during competitive sports and recreational activities. However, most of them have mild or no symptoms, and thus 50% of them go untreated (Collins et al., 1999). Actually, hospital-treated MTBIs are no more than 100–300/100,000 (Harmon et al., 2013). Neurological, cognitive and behavioral deficits, caused by MTBIs, are observed only for a short period of time. Headaches, vomiting, cognitive slowing, fatigue, dizziness, depression, and problems with attention and memory can be some of its symptoms (D’Hemecourt, 2011; Ruff, 2011; Sherer, Yablon & Nakase-Richardson, 2009). In the long run, MTBI can cause other post-concussive symptoms such as long-standing somatic symptoms, cognitive symptoms and affective symptoms (Holm et al., 2005). MTBI has shown a high rate of incidence, but it is difficult to detect the symptoms of one. To reveal the damaging mechanism and investigate a therapeutic method, previous studies on MTBI have made use of rat models. The problem is that a rat model is made through a very complicated process of anesthesia and surgery, such as craniotomy followed by the insertion of a plastic injury tube or single impact therapy or hydraulic induction of concussion (Sakurai et al., 2012; Redell et al., 2013; Dixon et al., 1987; Dixon et al., 1991). A recent study focused on tailoring their rat models of MTBI by considering the characteristics of MTBI, namely high-velocity and head acceleration (Kane et al., 2012). However, damaging mechanisms such as a shock to the head or surviving the impact from a fall cannot induce an MTBI alone. For another study, shocks were delivered to the craniums of rats equipped with helmet disks, but it was complicated to have the helmet disks put on (Xu et al., 2016). In the case of a method suggested by Tang et al. (1997), including an MTBI was comparatively simple and it did not cause skull fractures. However, their method caused brain edema that lasted about 48 h. The purpose of this study was to develop a tool that could artificially induce an MTBI in a safe and simple way and make the simulated MTBI equal in its damaging mechanism to a real MTBI. To confirm whether an MTBI had occurred, a behavioral test was conducted on experimental rats. Moreover, the tool was inspected for safety with magnetic resonance imaging (MRI) scans and blood tests. The safety inspection was focused on critical injuries such as a skull fracture or cerebral hemorrhage and stress that affected homeostasis.

## Materials and Methods

### Animal groups

Forty-four adult male Sprague-Dawley rats (200–250 g, 7 weeks-old) were divided into two groups: sham injury (*n* = 8), and mild traumatic brain injury (*n* = 36). The animals were maintained on a 12-h light/12-h dark cycle and at constant temperature range of 21–24 °C. Food and water were available ad libitum. All manipulation and experimental procedures on rats were approved by the local Ethics Committee (Ewha Medical Research Institute, No ESM 14-0252).

### Mild traumatic brain injury procedure

**Figure 1:**
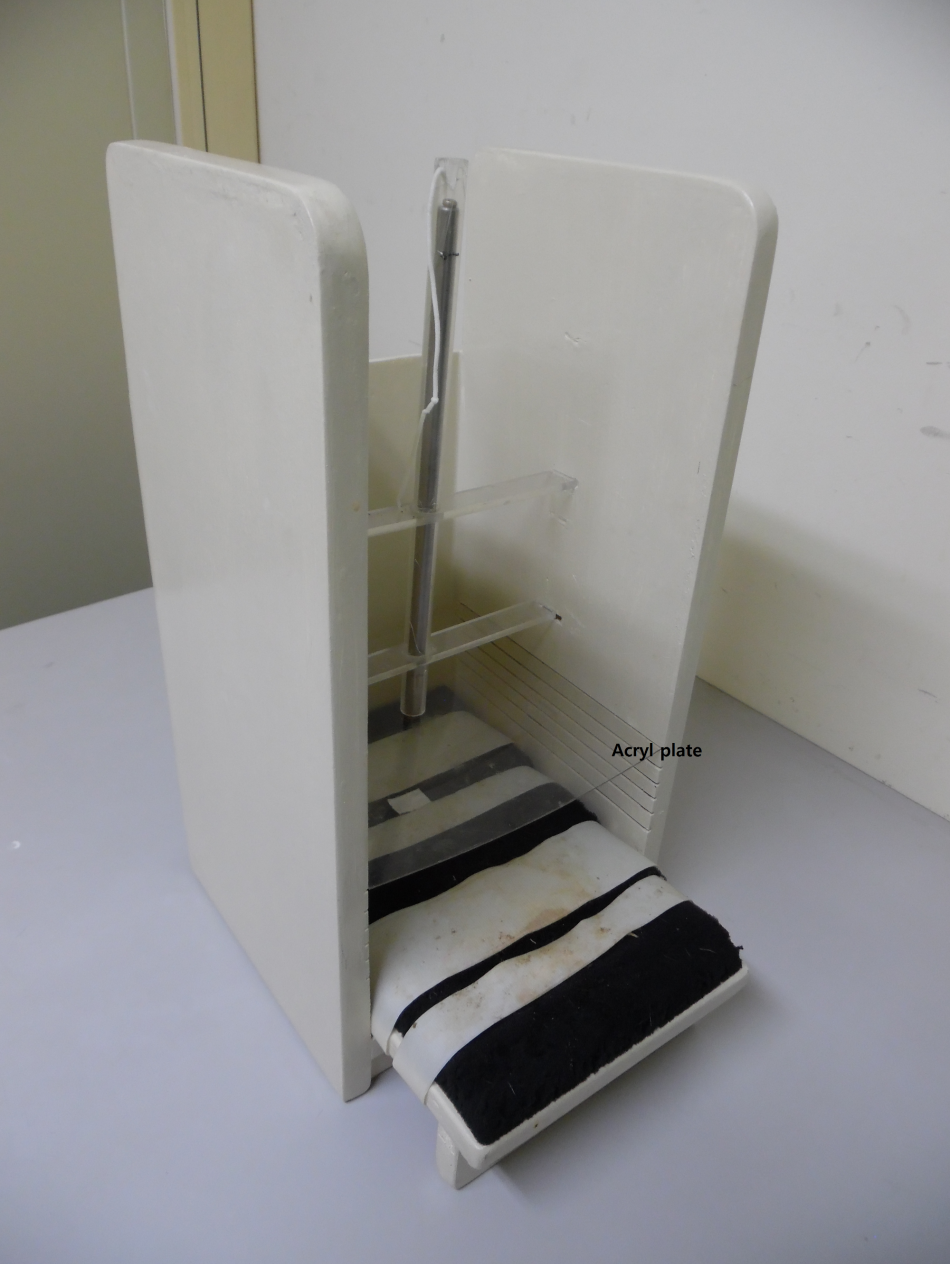
Device for mild traumatic brain injury using a rat as the subject. The components of the device are a vertical guide tube for the dropped weight and an acryl panel to absorb impact.

A weight drop device model was modified from a protocol originally developed for mice as described by Tang et al. (1997). Closed head MTBI was produced using a weight-loss device. We fixed a rat on the wooden plate (25 × 30 cm^2^) with Velcro, and a 175 g novel weight was dropped on the bregma of the rat. For decreasing risk of skull fracture, an acryl plate was placed above the head of the rat. The drop height (from top to acryl plate) was 30 cm, and the weight went through a polyvinyl chloride tube (inner diameter 11 cm, height 30 cm) to offer regular drop height. The plastic tube had small holes with regular intervals (2 cm) to reduce air resistance (Fig. 1). Sham injured animals were fixed on the wooden plate with Velcro for 5 s without further manipulation.

### Neurologic evaluation

#### Grid-walking and foot-fault test

Before the official test, the rats were pre-trained on a metal grid three times a day for three days. Grid-walking and foot-fault test were performed just after the MTBI. The apparatus consisted of an elevated 52 × 40 cm^2^ metal grid with grid cell of 3 × 3 cm^2^ (Barbosa et al., 2015). It was elevated 30 cm above the floor, and the metal grid was made of stainless steel. The rats were placed in the center square of the apparatus, and they were free to explore for 1 min. Behaviors in the grid were recorded with a video camera. During 1 min observation period, the following parameters were quantified: (a) total number of footsteps of hind limb, (b) number of foot fault, (c) foot fault ratio (number of foot faults/total number of footsteps), and (d) latency (time spent after being placed on the metal grid to move). Foot fault tests were performed before and 5 min after the MTBI.

#### Rota rod

Before the official test, rats were adapted to Rota rod three times a day for three days. The tests were performed before and 10 min after Grid-walking and foot-fault test. The specifications were for a Rota rod treadmill of a metal roller of diameter 40 mm, speed tachometer of 15 rpm. The rod was divided into five equal segments at 9 cm intervals. A rat was placed on the roller, and the time the rat stayed on it was measured (Tiwari et al., 2015).

### Magnetic resonance imaging and blood sampling

Magnetic resonance imaging (MRI) of mild traumatic brain injured group was performed 24 h after MTBI. The MRI confirmed presence of the skull fracture, brain hemorrhage and diffuse axonal injury. MRI scans were carried out with a four-element phased-array animal-dedicated with a 5-cm inner diameter surface coil (Chenguang Medical Technology, Co., Ltd., Shanghai, China). T2 weighted images were taken using a standard spin echo sequence (TE 22 ms; TR 650 ms; slice thickness 3.00 mm; matrix scan 512; FOV 100.00 mm). In addition, subclavian veins were punctured for blood samples to measure electrolytes, plasma glucose, and plasma calcium before as well as 5 and 20 min after MTBI. Electrolyte levels were measured using the EasyElectrolytes (Medica Corporation, Bedford, MA, USA). Plasma glucose and calcium levels were measured using BS-400 (Mindray, Shenzhen, China).

### Immunohistochemical assessment

One day after MTBI, 6 rats (three rats in the MTBI and three rats in the sham injury group) were euthanized and the brains were removed and fixed by immersion in 10% neutral buffered formalin solution. Three sections per group were then congruent compared to each other. Coronal sections at 4 µm thickness were prepared for planes having the level primary motor cortex with hippocampus. For immunohistochemical analysis, the sections were treated with primary antibody against glial fibrillary acidic protein (GFAP, rabbit polyclonal antibody 1:5,000 dilution, Abcam Ab7260) (Abcam, Cambridge, UK) at room temperature for 60 min. The sections were washed 3 times in TBS for 5 min each and incubated with conjugated secondary antibodies (Dako EnVision + System-HRP labeled anti-rabbit) (Dako, Glostrup, Denmark). The sections were then washed three times with TBS and incubated with diaminobenzidine (DAB) for 3–5 min. The region of interest (ROI) was the primary motor cortex (layers I–III) and CA1 of hippocampus. Integral intensity of GFAP expressions was measured using computer-assisted image analysis program (AnalySIS, Soft Imaging System, GmbH, Münster, Germany). Images were captured from two areas within the motor cortex and hippocampus. The software automatically converted all immune-labeled elements beyond the threshold range into pure red pixels and converted the rest of the image into pure blue pixels. The software then calculated the density of pure red pixels for statistical analysis.

### Statistical analysis

Comparisons of measurements between before and after MTBI were performed using paired-*t* test. Statistical analysis was performed using SPSS ver. 20.0 (IBM SPSS, Armonk, NY, USA) and *p*-values under 0.05 were considered significant. To analyze the change between before and after sham injury, a Wilcoxon singed rank test was used.

## Results

### Animal characteristics in magnetic resonance imaging

**Figure 2:**
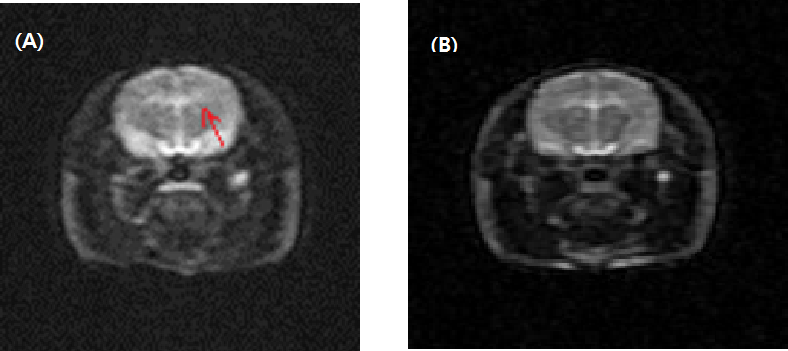
Magnetic resonance imaging after a mild traumatic brain injury. Arrows represent subarachnoid hemorrhage (A). There was no significant cerebral hemorrhage, intracranial hemorrhage, or diffuse axonal injury in 34 rats (B).

A total of 36 rats were applied to the concussion model. The MRI findings of 34 rats appeared normal. Only two rats had small amount of subarachnoid hemorrhage, which was not a fatal injury. There was no intracerebral hemorrhage, skull fracture, diffuse axonal injury or death (Table 1 and Fig. 2). The device took just 1–2 min to organize mild traumatic brain injury.

**Table 1:** Anatomical change after the mild traumatic brain injury (MTBI).

	Number
Total number of MTBI group	36
Mild traumatic brain injury	34
Subarachnoid hemorrhage	2
Intraventricular hemorrhage	0
Intracerebral hemorrhage	0
Skull fracture	0
Diffuse axonal injury	0

### Neurologic findings

#### Grid-walking and foot fault test

Thirty-four rats, appeared normal on the MRI, but showed a significant decrease in the total number of footsteps in the foot fault test (*p*-value < 0.001). The mean in the number of the footsteps was 40.12 ± 10.96 before MTBI, and 17.50 ± 14.50 after MTBI.

A decrease in different aspects of action was shown on the metal grid for 1 min. Foot fault steps before MTBI was 2.21 ± 1.61 per min, which had an error rate of 0.07 ± 0.09 in the total number of footsteps. After MTBI, however, the foot fault steps were decreased to 0.59 ± 0.74 per min (*p*-value < 0.001) with the decreased error rate of 0.04 ± 0.06 on the total number of footsteps (*p*-value = 0.01). Latency before MTBI was 0.94 ± 1.41, but it was prolonged to 5.26 ± 11.39 s after MTBI with statistical significance (*p*-value = 0.02). In the sham injury group, there were no significant changes of the total number of footsteps, the number of foot fault step, foot fault ratios and latency to move (Table 2).

**Table 2:** Comparison of grid walking test parameters before and after the mild traumatic brain injury with sham injury.

	Total foot step (number/min)	Foot fault step (number/min)	Foot fault ratio	Latency (s)
MTBI				
Before MTBI	40.12 ± 10.96	2.21 ± 1.61	0.07 ± 0.09	0.94 ± 1.41
After MTBI	17.50 ± 14.50	0.59 ± 0.74	0.04 ± 0.06	5.26 ± 11.39
*p*-value	<0.001	<0.001	0.01	0.02
Sham injury				
Before sham injury	35.50 ± 16.03	1.00 ± 1.07	0.02 ± 0.03	1.00 ± 1.60
After sham injury	31.88 ± 14.38	0.38 ± 0.74	0.02 ± 0.02	0.75 ± 1.75
*p*-value	0.31	0.13	0.59	0.72

**Notes.**

Values are mean ± standard deviation.

MTBI Mild traumatic brain injury s Second Min Minute

#### Rota rod test

For the Rota rod evaluation, the rats maintained rolling with balance for 10.00 ± 11.21 s before MTBI. They were able to keep rolling for 13.97 ± 16.09 s after MTBI, thus there was no significant difference in Rota rod results between before and after MTBI. In the sham injury group, animals stayed on the roller for 25.13 ± 16.62 s before and 34.13 ± 11.66 s after the sham injury (*p*-value > 0.05).

### Blood biochemical findings

There is no significant difference between before and after MTBI and the sham injury group in blood test. The sodium level showed no significant difference between before and after MTBI and for the sham injury (before MTBI 138.34 ± 3.33 mmol/L; after MTBI 138.45 ± 1.88 mmol/L; before sham injury 138.38 ± 0.67 mmol/L; after sham injury 138.64 ± 0.98 mmol/L). Similarly, for the potassium level changes, there were no significant changes (before MTBI 5.38 ± 0.17 mmol/L; after MTBI 4.98 ±  0.11 mmol/L; before sham injury 4.74 ± 0.36 mmol/L; after MTBI 4.41 ± 0.54 mmol/L). A decrease in serum glucose level was detected from 210.77 ± 49.33 mg/dL before MTBI to 196.00 ± 42.04 mg/dL after MTBI, but this result was not statistically significant. Also, a calcium level change in the MTBI group was also detected from 1.17 ± 0.27 to 1.21 ±  0.17 mmol/L, but that was not statistically significant as well. The serum glucose and calcium levels had not changed in the sham injury group (Table 3).

**Table 3:** Comparison of serum parameters before and after the mild traumatic brain injury with sham injury.

	Sodium (mmol/L)	Potassium (mmol/L)	Glucose (mg/dL)	Calcium (mmol/L)
MTBI				
Before MTBI	138.34 ± 3.33	5.38 ± 0.17	210.77 ± 49.33	1.17 ± 0.27
After MTBI	138.45 ± 1.88	4.98 ± 0.11	196.00 ± 42.04	1.21 ± 0.17
*p*-value	0.87	0.65	0.06	0.51
Sham injury				
Before sham injury	138.38 ± 0.67	4.74 ± 0.36	191.38 ± 16.69	0.95 ± 0.27
After sham injury	138.34 ± 0.98	4.41 ± 0.54	188.00 ± 16.23	0.98 ± 0.25
*p*-value	0.50	0.13	0.18	0.58

**Notes.**

Values are mean ± standard deviation.

MTBI Mild traumatic brain injury s Second Min Minute

**Figure 3:**
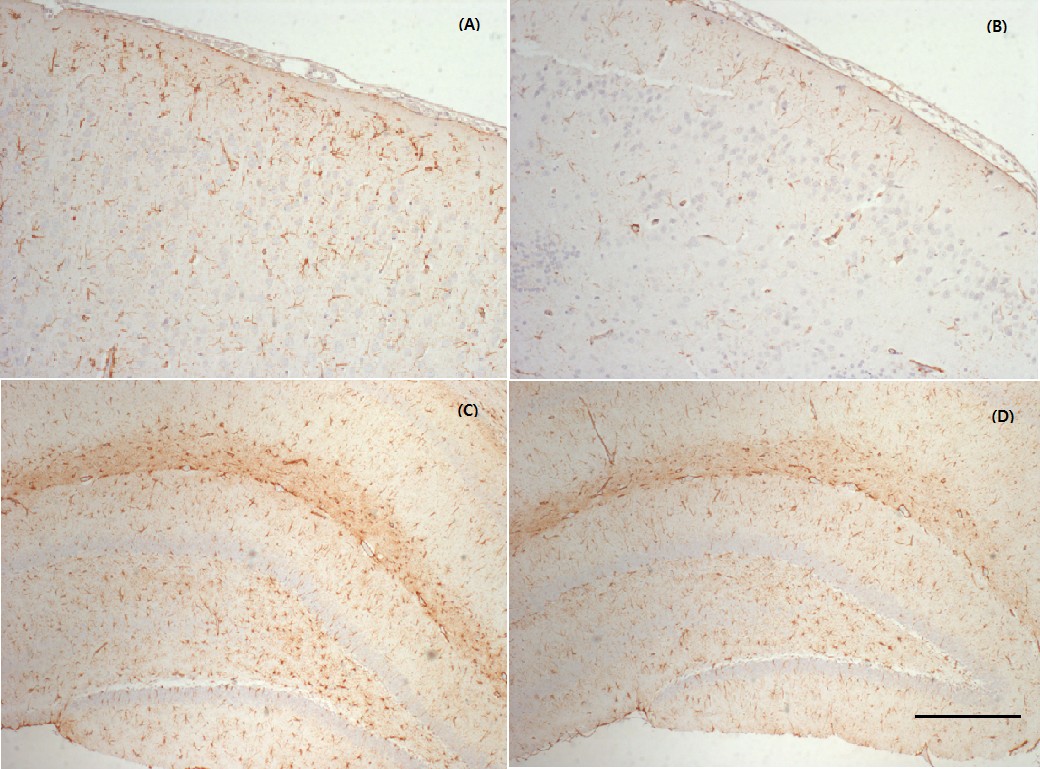
Representative photographs of the cerebral cortex and hippocampus samples showing GFAP expression. MTBI led to a slightly increased GFAP expression in the cortex (A) and hippocampus (C) compared with controls (B, D). The calibration bar represents 200 µm scale.

### Expression of GFAP immunoreactivity

**Figure 4:**
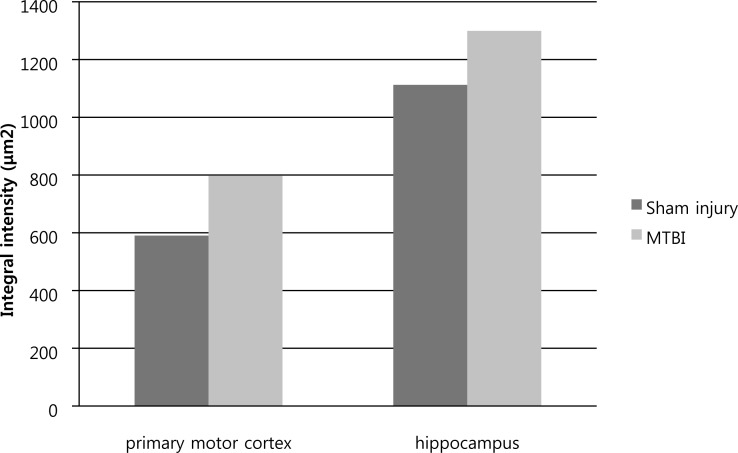
GFAP immunoreactivity analysis. Immunohistochemical analysis of GFAP in primary motor cortex and hippocampus is increased in the mid traumatic brain injury (MTBI) group compared to the sham injury group.

Immunohistochemical determination of GFAP was performed to determine reactive astrocytosis. Figure 3 is a representative immunofluorescent image which shows slightly increased immunoreactivity of GFAP in the cerebral cortex around injured bregma and hippocampus. The integral intensity of GFAP expression in the MTBI group (798.03 ± 93.42 in cortex, 1299.35 ± 154.31 in hippocampus) increased in comparison with control group (590.28 ± 137.20 in cortex, 1112.42 ± 123.27) in hippocampus (Fig. 4). The GFAP immunoreactivity showed that increased expression in the cortex and hippocampus in the MTBI group, which was higher than in the control group, but this was not significant.

## Discussion

This study aimed to introduce a new MTBI model that is similar to the damaging mechanism of MTBI. The implementation was a modification of the method suggested by Tang et al. (1997) that used a weight to deliver a shock to the head for the rat animal model. Because the physical impact, as suggested by Tang et al., was judged to be too big in that model, we reduce the drop height and placed an acryl sheet between the rat head and the weight so that some of the impact shock could be absorbed. The study of Tang et al. focused on presence of only skull fractures, and did not describe a serious outcome such as a brain hemorrhage. In this regard, the side effects seen our method may be compared to the study by Kane et al. that have been used in many studies. In their study, a 95-gram weight was dropped at a height of 1 meter onto a foil on which a rat stayed. The shock from the weight made the rat fall onto a sponge cushion that was 10 centimeters below the foil, and that induced a high-velocity with resultant head acceleration. The method has widely been used to make a tool that causes MTBI in rats (Kane et al., 2012). In the study of Kane et al., skull fractures, intracranial bleeding, respiratory arrests and seizures occurred in 10% of rats, and the mortality rate reached 5%, with the method thus far being recognized as safe. On the other hand, in our study, a subarachnoid hemorrhage occurred just in 5.5% of cases (two out of 36 animals). In addition, no mortality among the test animals occurred. These results imply that method can be safer than other methods; in addition, our MTBI model can be conducted within 1 or 2 min and does not need an incision and surgery, unlike previous studies (Sakurai et al., 2012; Redell et al., 2013; Dixon et al., 1987; Dixon et al., 1991).

The grid-walking/foot-fault test is known to assay for sensorimotor coordination in neurological diseases such as a cerebral infarction, cortex injury, and Parkinson’s disease that may be affected by motor ability (Zhang et al., 2002; Barth, Jones & Schallert, 1990; Shanina et al., 2006; Chao et al., 2012). In our study, with the injury method developed herein, MTBI characteristics were seen in rats with a behavioral delay in the grid-walking and foot fault test. In several cases, no movements were observed in the initial one minute of the test. A delay in latency results from a temporary unconsciousness that occurs after an MTBI, or may be due to post-concussive symptoms such as a headache, dizziness, or irritability. From induction of MTBI, rats became slow in movement on the metal grid, considerably reducing the number of their steps in addition to latency, along with many rats lying almost motionless. These seem to be an aspect of alterations in plasticity and activation and from hypometabolism as in the study of Shrey, Griesbach & Giza (2011). Reductions in foot-fault steps and foot-fault error rates do not result from improvement in sensorimotor coordination after an MTBI but are more likely to be caused by a reduction in real movements.

The Rota rod test was to examine balance impairments. The test detected the problems of balance and postural equilibriums subsequent to MTBI (Guskiewicz, 2011). In this study, there was no statistical significant difference before and after MTBI regarding the Rota rod rolling duration. It was probably because the Rota rod test was conducted after the grid-walking and foot-fault test, and that the rats recovered from an MTBI faster, as they were less injured than those in the other studies. It should be noted that the rats were fidgety after getting an MTBI and tended to have difficulty rolling, but these measurements could not be conducted.

By histological analysis, we could also reveal a mild injury at the cellular level. Immunohistochemical analyses confirmed increased GFAP expression, which indicative of a mild astrocytic response to injury (increased astrocytic activation) to injury. However, this was not significant as the number of stained rats was small and the time of sacrifice was too early. Giza & Hovda (2014) reported that later time points (four days to one month) showed maximal staining for gliosis (GFAP-glial fibrillary acidic protein).

Pathophysiological studies of MTBI have been carried out with various specimens including cerebrospinal fluids, brain cells and sera, with the studies indicating an efflux of potassium ions into the extracellular fluid, and an influx of sodium ions into the interior of cells in the acute phase of the response. The cellular depolarization temporarily causes the disruption of cell homeostasis, and increases the level of intracellular calcium ions (Clifton, Ziegler & Grossman, 1981; Domínguez & Raparla, 2014; Giza & Hovda, 2014). During post-trauma catecholamine is also released and glycolysis occurs (Clifton, Ziegler & Grossman, 1981; Shrey, Griesbach & Giza, 2011). In this study, the levels of serum electrolytes, glucose and calcium were measured 20 min after the induction of MTBI. As in previous studies, calcium accumulation occurred but it was not a statistically significant change. Given that this was measured with serum, it is presumed that calcium accumulation was only marginal. According to the report of Giza & Hovda (2001) glycolysis reaches its peak 6 min after the induction of MTBI, and hyperglycolysis ends 20 min later, and for 24 h afterwards, the cerebral glucose metabolism slows down. In this study, a blood test was conducted 20 min after the induction of MTBI, and thus hyperglycolysis could not be observed. In addition, although a change occurs in the electrical charge of the membrane, the imbalance of electrolytes in real blood was not observed due to homeostasis.

This study has several limitations. First, hyperglycolysis could not be analyzed because the blood test was conducted 20 min after the induction of MTBI and as not to influence behavioral measurements. Second, the number of experimental rats is considered to be small for the study. It is possible, of course, to generalize the rat models of MTBI with the use of parametric statistics, yet a larger number of experimental rats might be helpful in raising the validity and reliability of the findings of this study. Third, the above results only confirmed the early effect of the MTBI model. The long-term effect on behavior, learning and memory was not revealed. A long-term study would be required to prove the severity of MTBI by the device and its chronic effect. Fourth, with the small number of test animals in the sham injury group, it was hard to compare the measurements with MTBI group directly.

This study allowed testing a rat model of MTBI and to demonstrate its safety and simplicity. The traumatic brain injuries induced in this study were much milder than those in other studies. This type of modeling of MTBI is expected to allow studying pathophysiology or progression in the injury model as 50% of patients with MTBI do not visit a hospital on the perception of having only mild symptoms, thus making it difficult to assess their cases at early stages of post trauma. Furthermore, due to its characteristics, this type of model can be applied and planned for studies of repetitive MTBI.

##  Supplemental Information

10.7717/peerj.2818/supp-1 Supplemental Information 1 Rat concussion model: behavior, laboratory

10.7717/peerj.2818/supp-2 Data S1 Raw data (rats 1 to 10) of magnetic resonance imaging after a mild traumatic brain injury applied to confirm absence of gross brain lesion to prepare for Table 1 and Fig. 2

10.7717/peerj.2818/supp-3 Data S2 Raw data (rats 11 to 20) of magnetic resonance imaging after a mild traumatic brain injury applied to confirm absence of gross brain lesion to prepare for Table 1 and Fig. 2

10.7717/peerj.2818/supp-4 Data S3 Raw data (rats 21 to 34) of magnetic resonance imaging after a mild traumatic brain injury applied to confirm absence of gross brain lesion to prepare for Table 1 and Fig. 2

10.7717/peerj.2818/supp-5 Data S4 Raw data of magnetic resonance imaging after a mild traumatic brain injury applied to confirm presence of gross brain lesion to prepare for Table 1 and Fig. 2

## Additional Information and Declarations

### Data Availability

The following information was supplied regarding data availability: The raw data has been supplied as Data S1–S4.
