# Supplementary figures and images for: A simple rat model of mild traumatic brain injury: a device to reproduce anatomical and neurological changes of mild traumatic brain injury (part 7 of 7)
Source: PeerJ. 2017 Jan 3;5:e2818. doi: 10.7717/peerj.2818 (PMC5214841; doi:10.7717/peerj.2818)

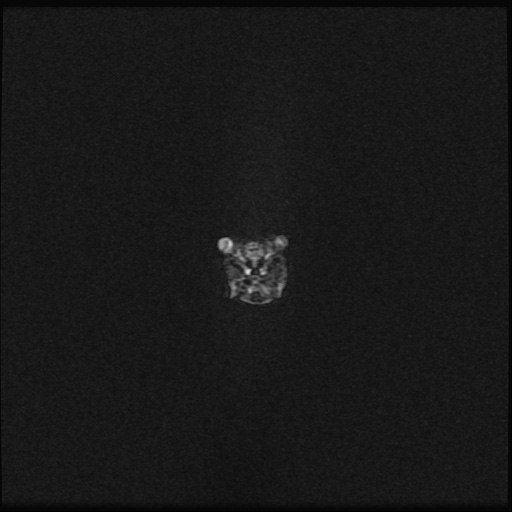

Supplement: Data S4 [file peerj-05-2818-s005.zip › rat1/I0000000.jpg]

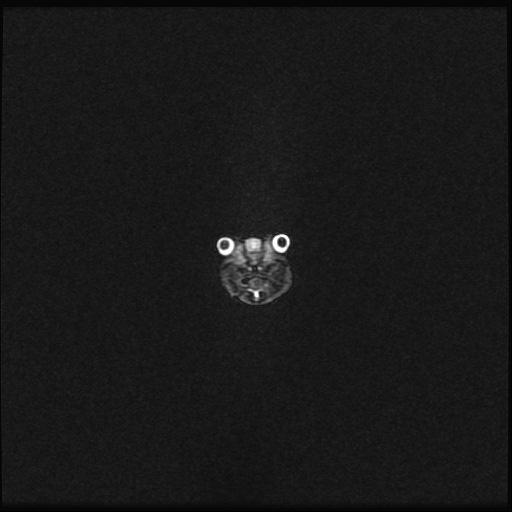

Supplement: Data S4 [file peerj-05-2818-s005.zip › rat1/I0000001.jpg]

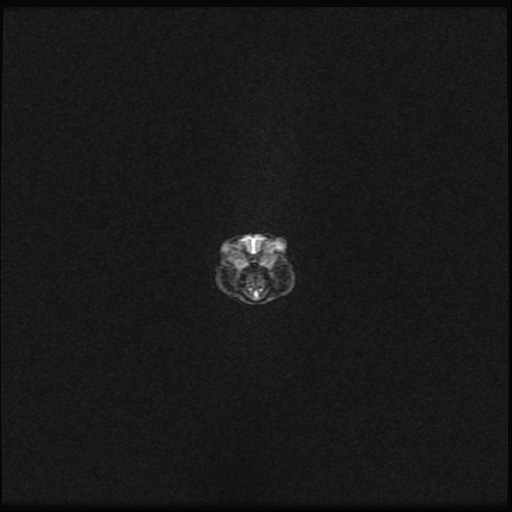

Supplement: Data S4 [file peerj-05-2818-s005.zip › rat1/I0000002.jpg]

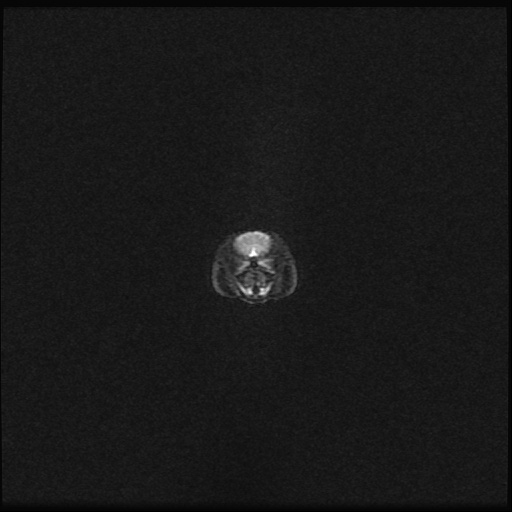

Supplement: Data S4 [file peerj-05-2818-s005.zip › rat1/I0000003.jpg]

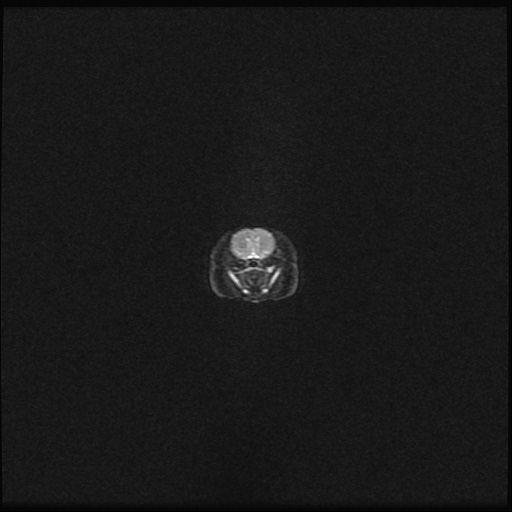

Supplement: Data S4 [file peerj-05-2818-s005.zip › rat1/I0000004.jpg]

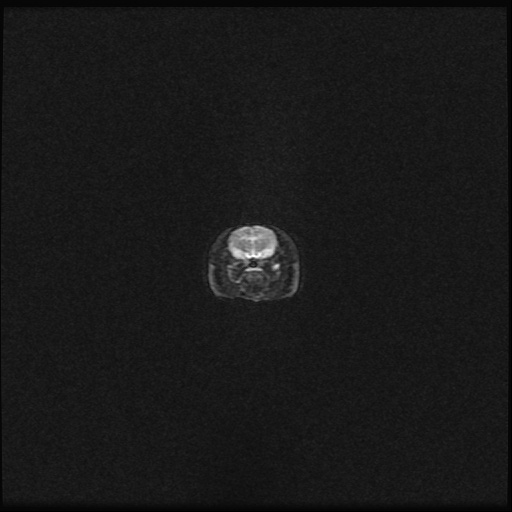

Supplement: Data S4 [file peerj-05-2818-s005.zip › rat1/I0000005.jpg]

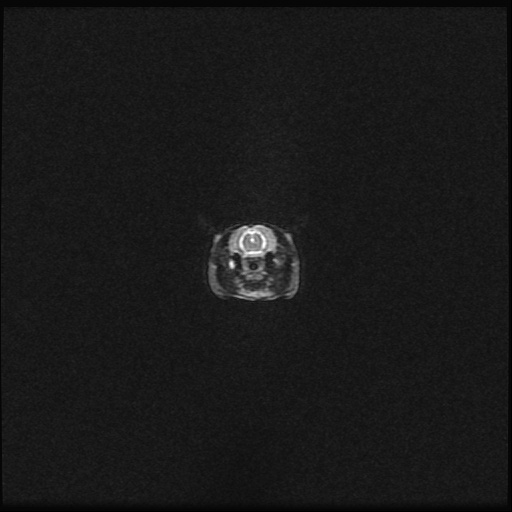

Supplement: Data S4 [file peerj-05-2818-s005.zip › rat1/I0000006.jpg]

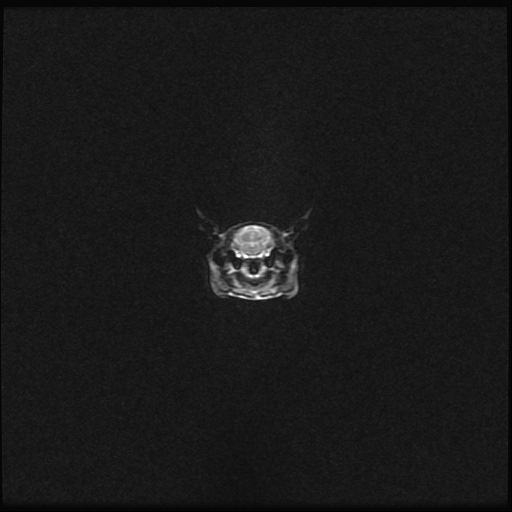

Supplement: Data S4 [file peerj-05-2818-s005.zip › rat1/I0000007.jpg]

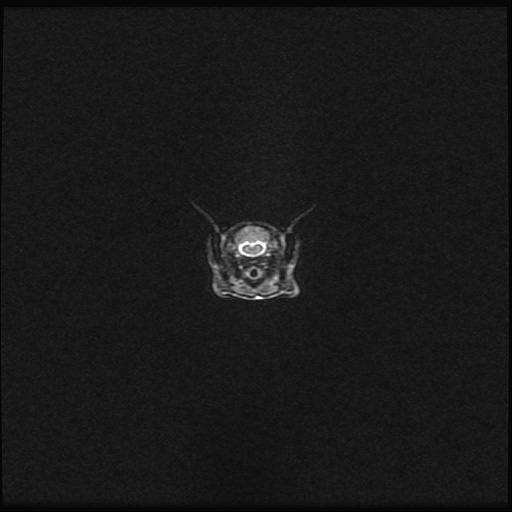

Supplement: Data S4 [file peerj-05-2818-s005.zip › rat1/I0000008.jpg]

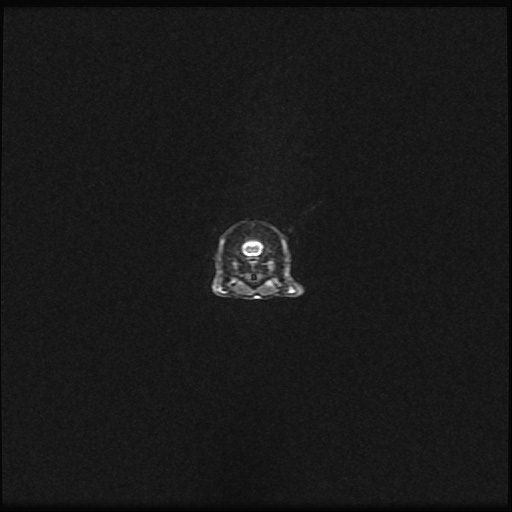

Supplement: Data S4 [file peerj-05-2818-s005.zip › rat1/I0000009.jpg]

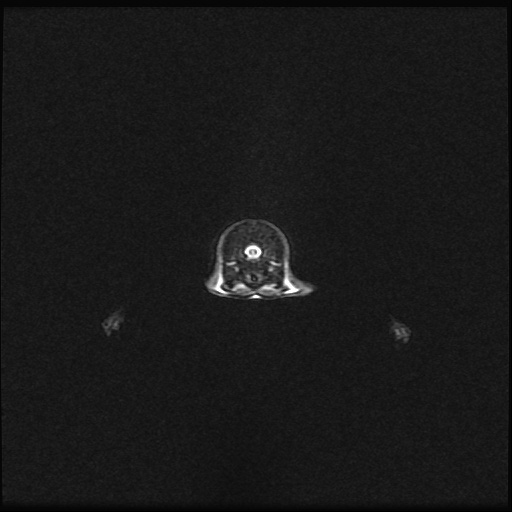

Supplement: Data S4 [file peerj-05-2818-s005.zip › rat1/I0000010.jpg]

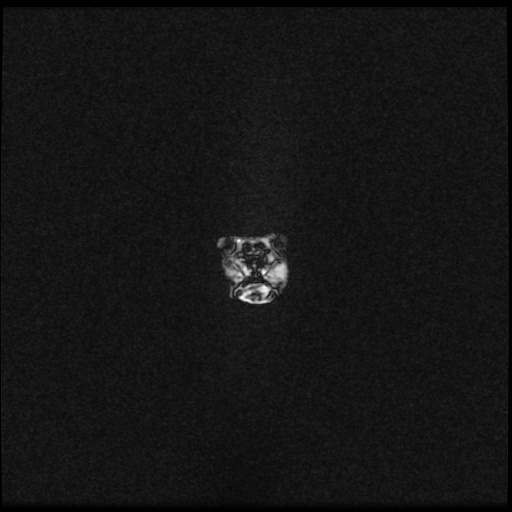

Supplement: Data S4 [file peerj-05-2818-s005.zip › rat1/I0000011.jpg]

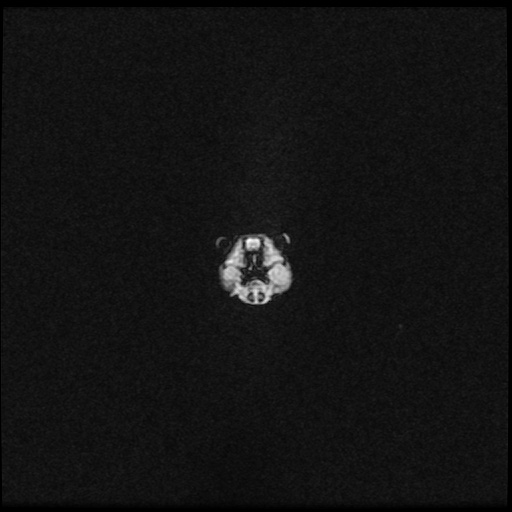

Supplement: Data S4 [file peerj-05-2818-s005.zip › rat1/I0000012.jpg]

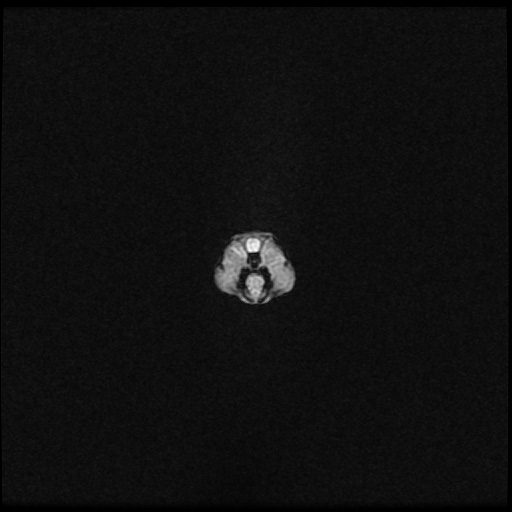

Supplement: Data S4 [file peerj-05-2818-s005.zip › rat1/I0000013.jpg]

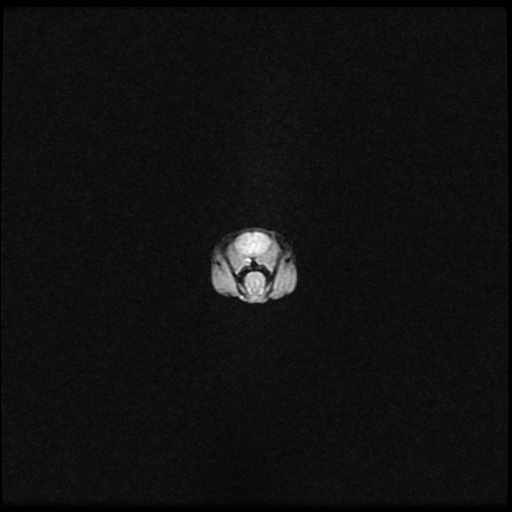

Supplement: Data S4 [file peerj-05-2818-s005.zip › rat1/I0000014.jpg]

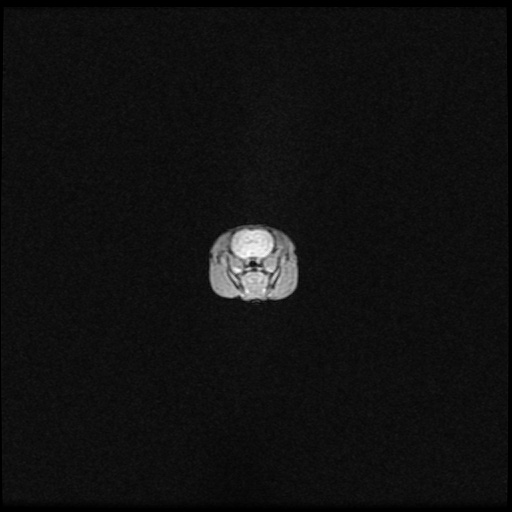

Supplement: Data S4 [file peerj-05-2818-s005.zip › rat1/I0000015.jpg]

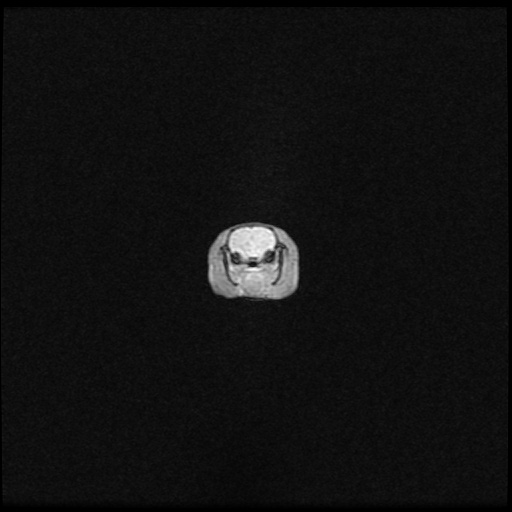

Supplement: Data S4 [file peerj-05-2818-s005.zip › rat1/I0000016.jpg]

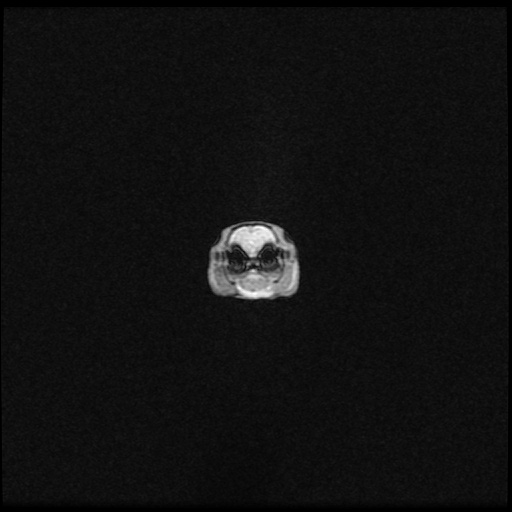

Supplement: Data S4 [file peerj-05-2818-s005.zip › rat1/I0000017.jpg]

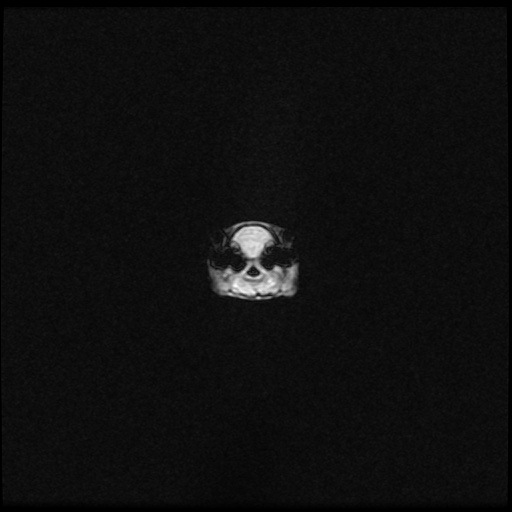

Supplement: Data S4 [file peerj-05-2818-s005.zip › rat1/I0000018.jpg]

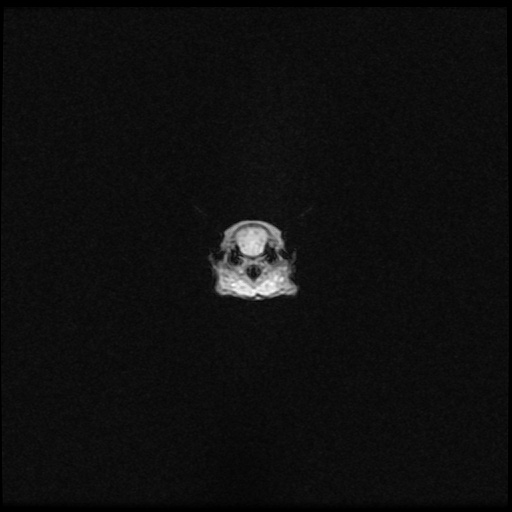

Supplement: Data S4 [file peerj-05-2818-s005.zip › rat1/I0000019.jpg]

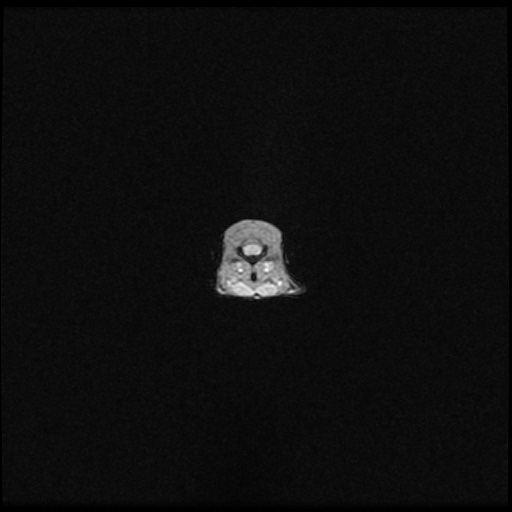

Supplement: Data S4 [file peerj-05-2818-s005.zip › rat1/I0000020.jpg]

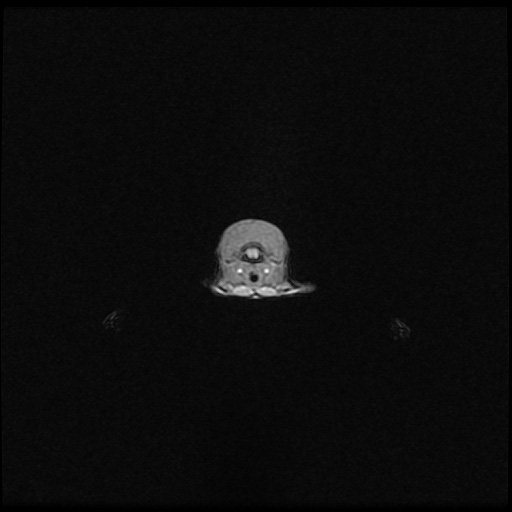

Supplement: Data S4 [file peerj-05-2818-s005.zip › rat1/I0000021.jpg]

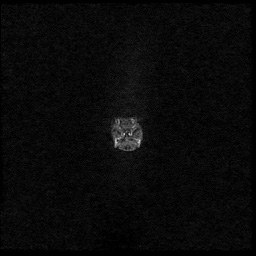

Supplement: Data S4 [file peerj-05-2818-s005.zip › rat1/I0000022.jpg]

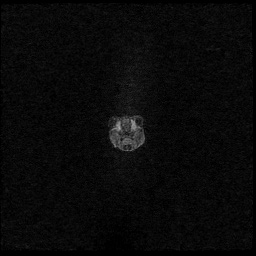

Supplement: Data S4 [file peerj-05-2818-s005.zip › rat1/I0000023.jpg]

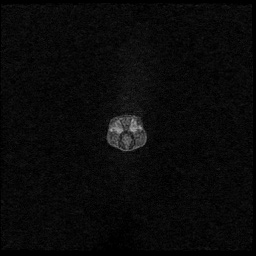

Supplement: Data S4 [file peerj-05-2818-s005.zip › rat1/I0000024.jpg]

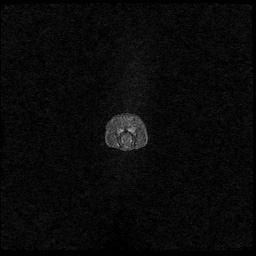

Supplement: Data S4 [file peerj-05-2818-s005.zip › rat1/I0000025.jpg]

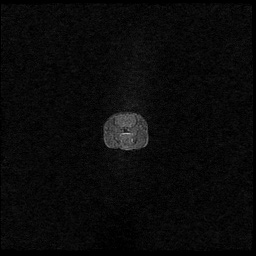

Supplement: Data S4 [file peerj-05-2818-s005.zip › rat1/I0000026.jpg]

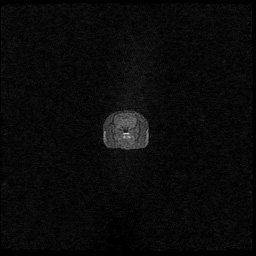

Supplement: Data S4 [file peerj-05-2818-s005.zip › rat1/I0000027.jpg]

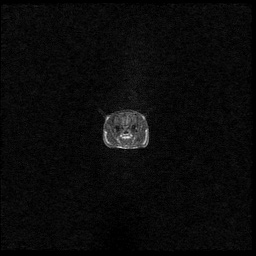

Supplement: Data S4 [file peerj-05-2818-s005.zip › rat1/I0000028.jpg]

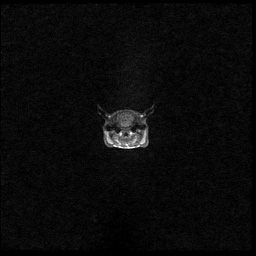

Supplement: Data S4 [file peerj-05-2818-s005.zip › rat1/I0000029.jpg]

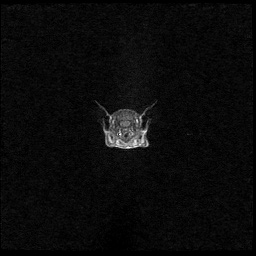

Supplement: Data S4 [file peerj-05-2818-s005.zip › rat1/I0000030.jpg]

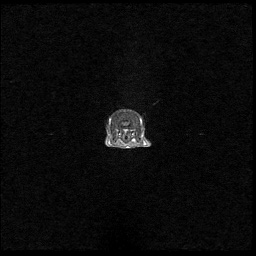

Supplement: Data S4 [file peerj-05-2818-s005.zip › rat1/I0000031.jpg]

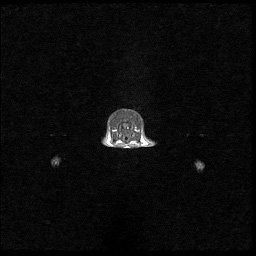

Supplement: Data S4 [file peerj-05-2818-s005.zip › rat1/I0000032.jpg]

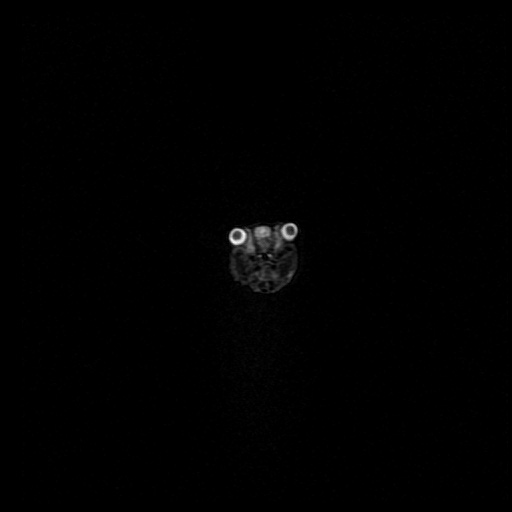

Supplement: Data S4 [file peerj-05-2818-s005.zip › rat2/I0000000.jpg]

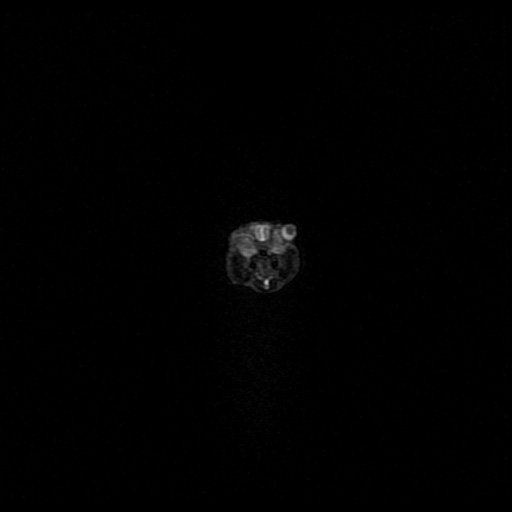

Supplement: Data S4 [file peerj-05-2818-s005.zip › rat2/I0000001.jpg]

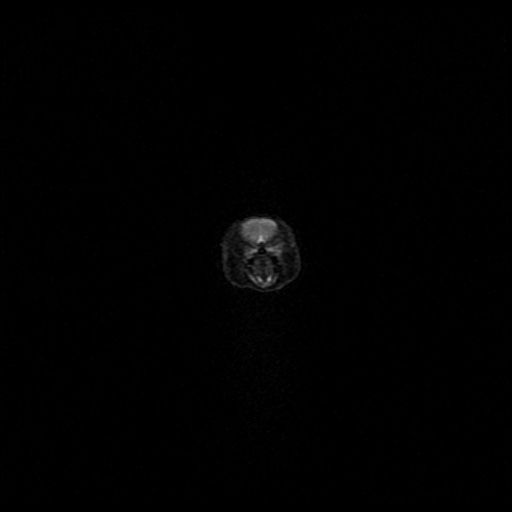

Supplement: Data S4 [file peerj-05-2818-s005.zip › rat2/I0000002.jpg]

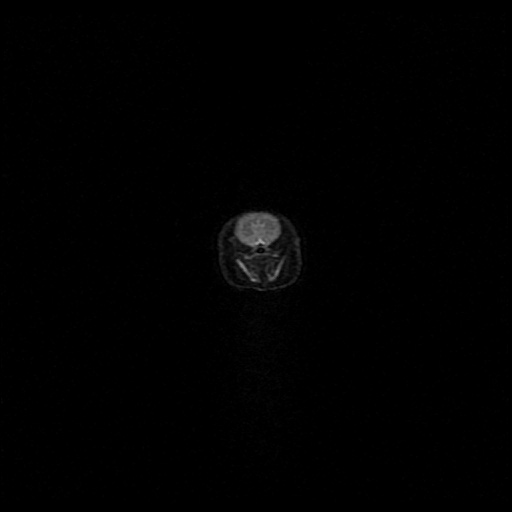

Supplement: Data S4 [file peerj-05-2818-s005.zip › rat2/I0000003.jpg]

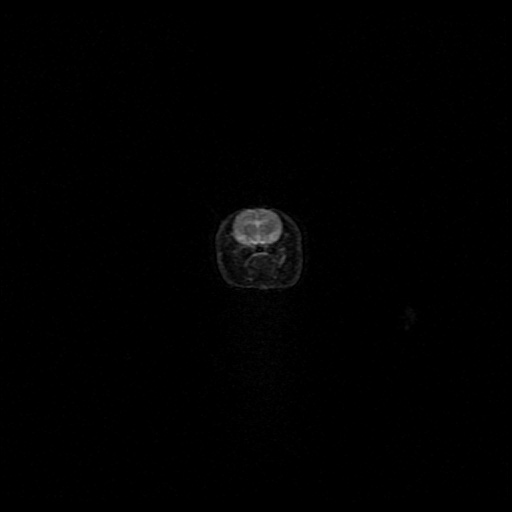

Supplement: Data S4 [file peerj-05-2818-s005.zip › rat2/I0000004.jpg]

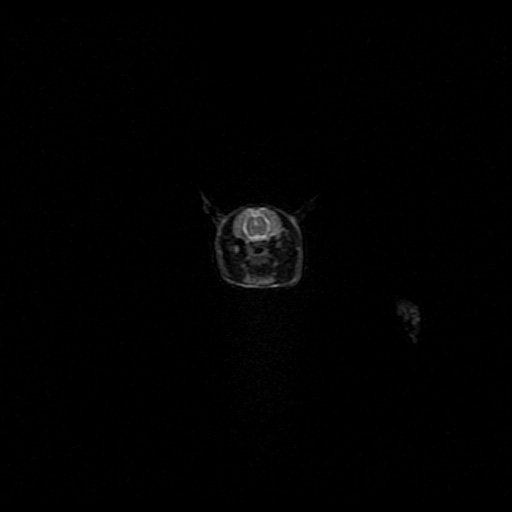

Supplement: Data S4 [file peerj-05-2818-s005.zip › rat2/I0000005.jpg]

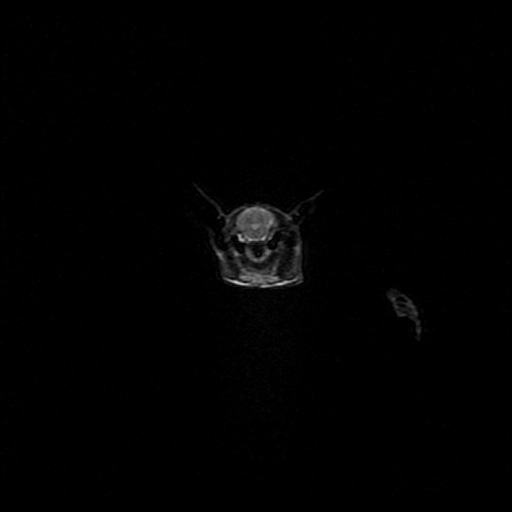

Supplement: Data S4 [file peerj-05-2818-s005.zip › rat2/I0000006.jpg]

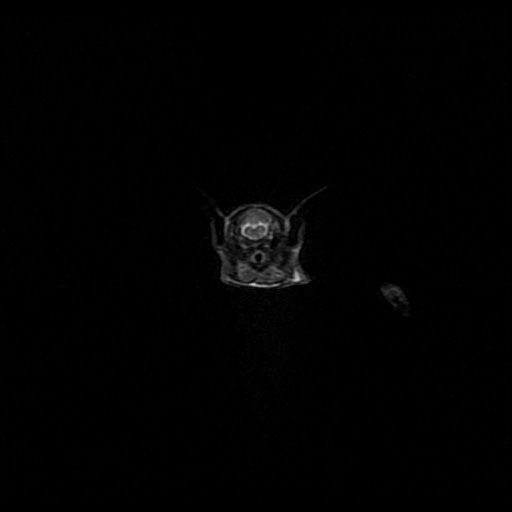

Supplement: Data S4 [file peerj-05-2818-s005.zip › rat2/I0000007.jpg]

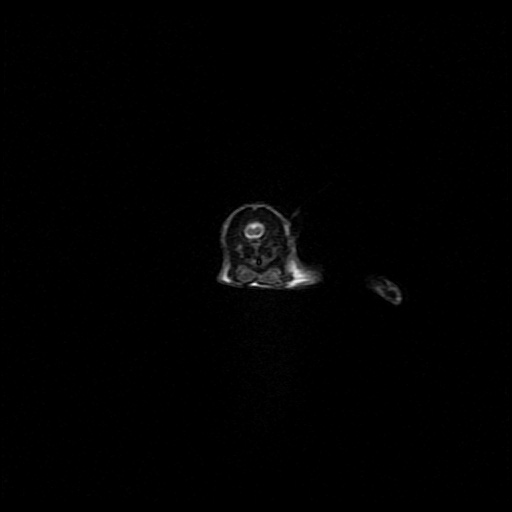

Supplement: Data S4 [file peerj-05-2818-s005.zip › rat2/I0000008.jpg]

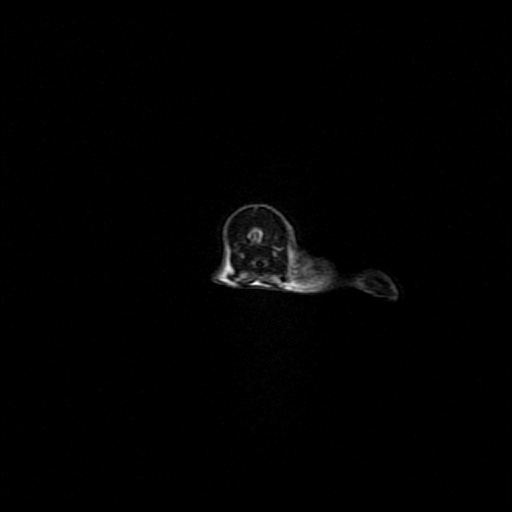

Supplement: Data S4 [file peerj-05-2818-s005.zip › rat2/I0000009.jpg]

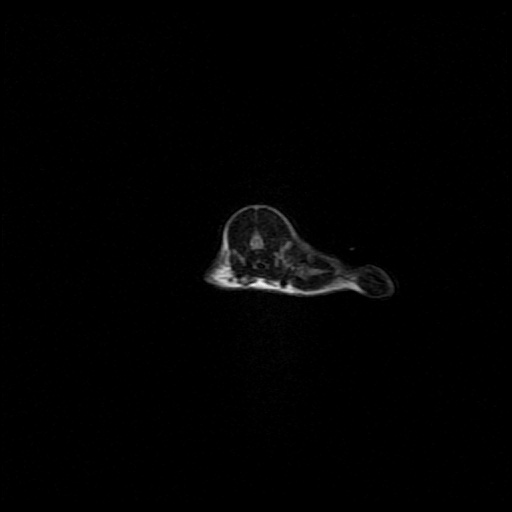

Supplement: Data S4 [file peerj-05-2818-s005.zip › rat2/I0000010.jpg]

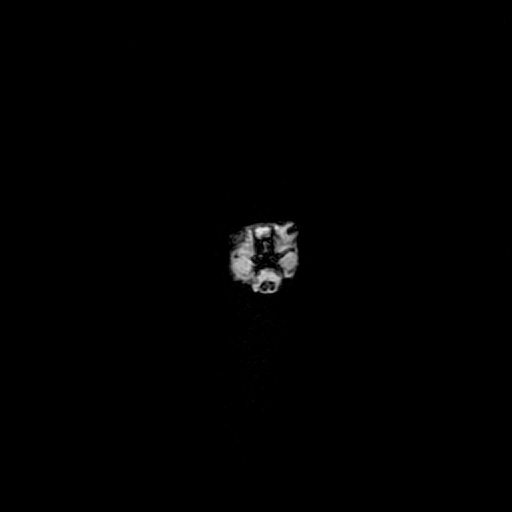

Supplement: Data S4 [file peerj-05-2818-s005.zip › rat2/I0000011.jpg]

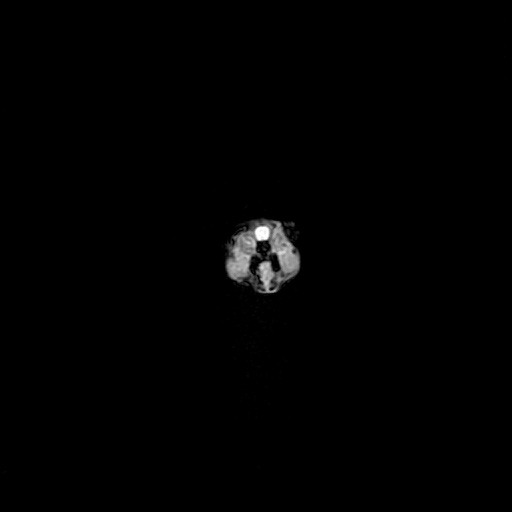

Supplement: Data S4 [file peerj-05-2818-s005.zip › rat2/I0000012.jpg]

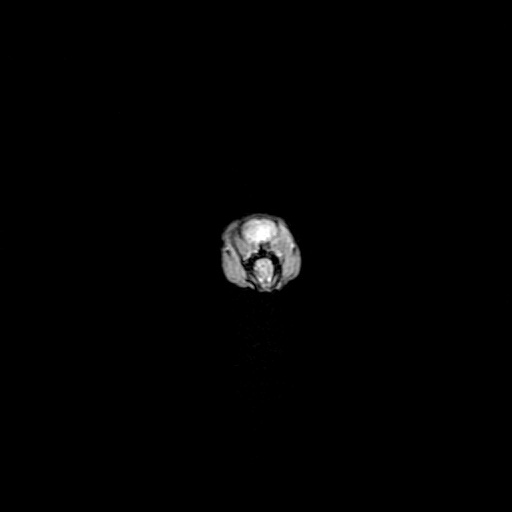

Supplement: Data S4 [file peerj-05-2818-s005.zip › rat2/I0000013.jpg]

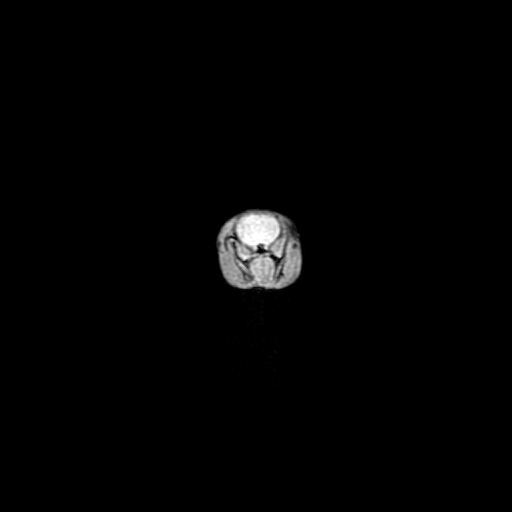

Supplement: Data S4 [file peerj-05-2818-s005.zip › rat2/I0000014.jpg]

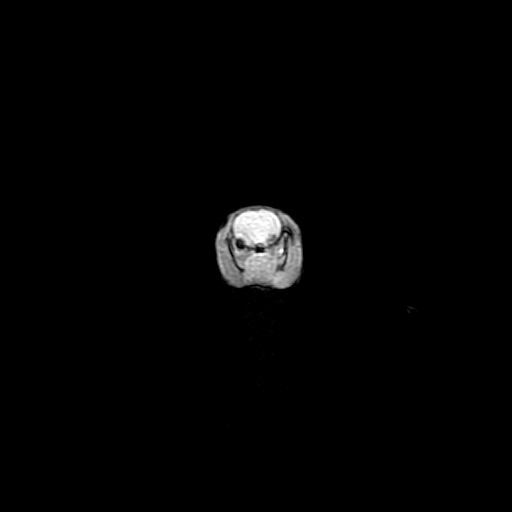

Supplement: Data S4 [file peerj-05-2818-s005.zip › rat2/I0000015.jpg]

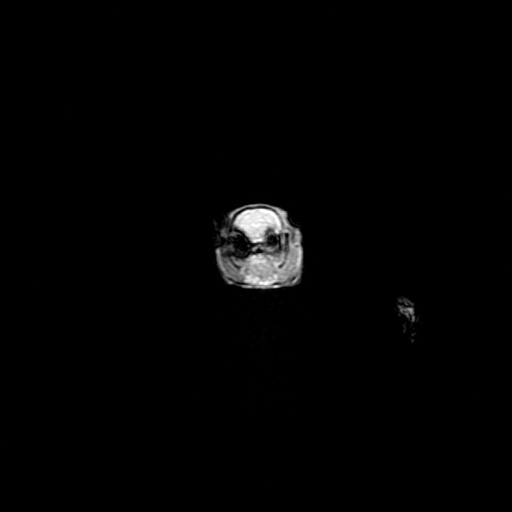

Supplement: Data S4 [file peerj-05-2818-s005.zip › rat2/I0000016.jpg]

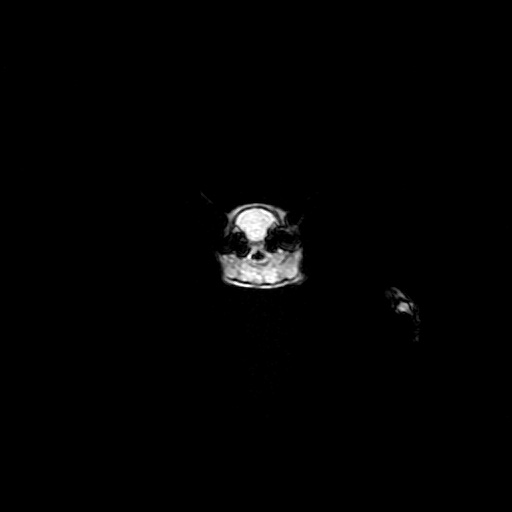

Supplement: Data S4 [file peerj-05-2818-s005.zip › rat2/I0000017.jpg]

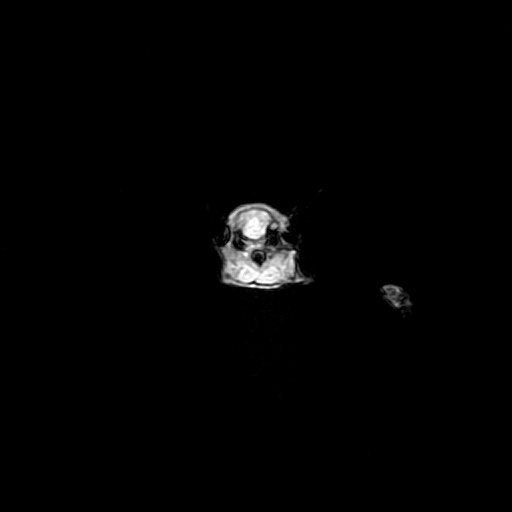

Supplement: Data S4 [file peerj-05-2818-s005.zip › rat2/I0000018.jpg]

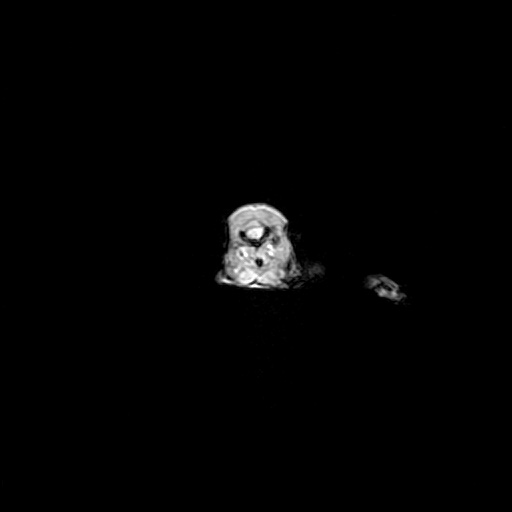

Supplement: Data S4 [file peerj-05-2818-s005.zip › rat2/I0000019.jpg]

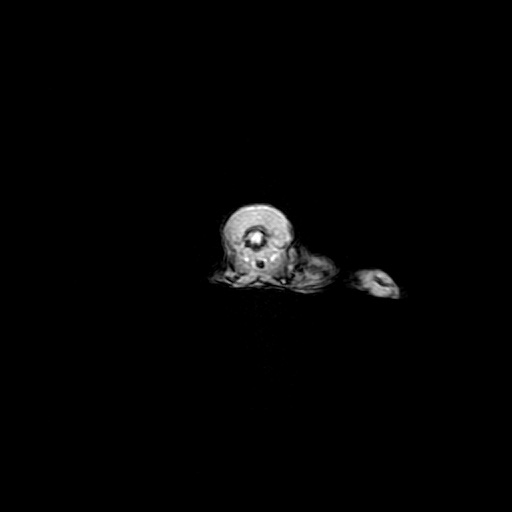

Supplement: Data S4 [file peerj-05-2818-s005.zip › rat2/I0000020.jpg]

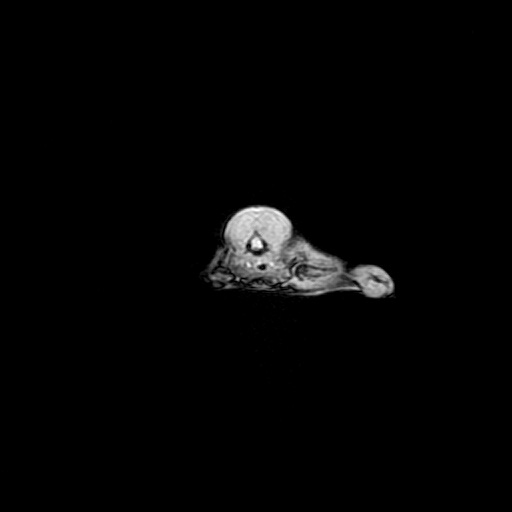

Supplement: Data S4 [file peerj-05-2818-s005.zip › rat2/I0000021.jpg]

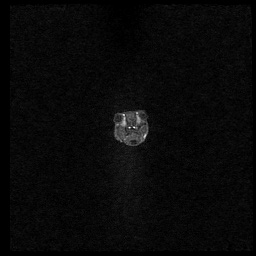

Supplement: Data S4 [file peerj-05-2818-s005.zip › rat2/I0000022.jpg]

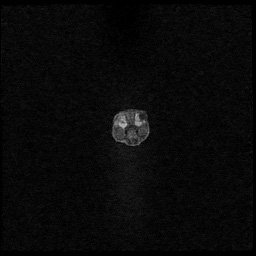

Supplement: Data S4 [file peerj-05-2818-s005.zip › rat2/I0000023.jpg]

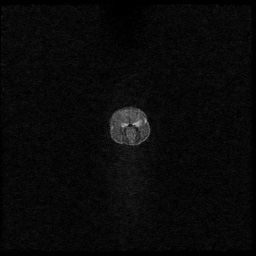

Supplement: Data S4 [file peerj-05-2818-s005.zip › rat2/I0000024.jpg]

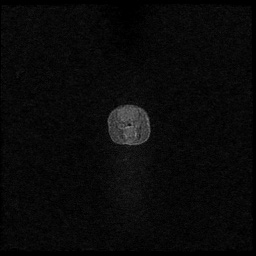

Supplement: Data S4 [file peerj-05-2818-s005.zip › rat2/I0000025.jpg]

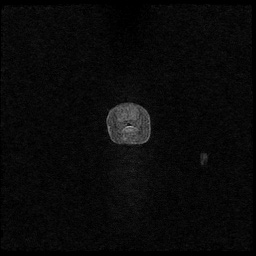

Supplement: Data S4 [file peerj-05-2818-s005.zip › rat2/I0000026.jpg]

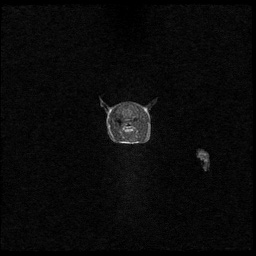

Supplement: Data S4 [file peerj-05-2818-s005.zip › rat2/I0000027.jpg]

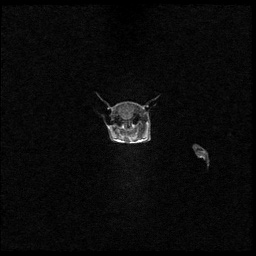

Supplement: Data S4 [file peerj-05-2818-s005.zip › rat2/I0000028.jpg]

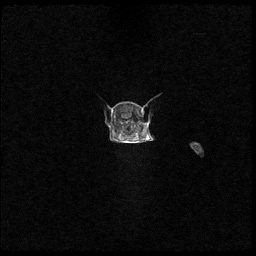

Supplement: Data S4 [file peerj-05-2818-s005.zip › rat2/I0000029.jpg]

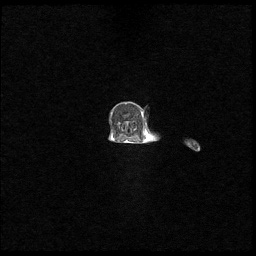

Supplement: Data S4 [file peerj-05-2818-s005.zip › rat2/I0000030.jpg]

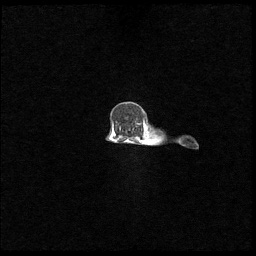

Supplement: Data S4 [file peerj-05-2818-s005.zip › rat2/I0000031.jpg]

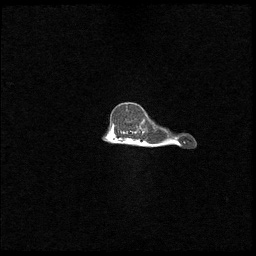

Supplement: Data S4 [file peerj-05-2818-s005.zip › rat2/I0000032.jpg]
